# Aging-Related Phenotypic Conversion of Medullary Microglia Enhances Intraoral Incisional Pain Sensitivity

**DOI:** 10.3390/ijms21217871

**Published:** 2020-10-23

**Authors:** Daisuke Ikutame, Kentaro Urata, Tatsuki Oto, Shintaro Fujiwara, Toshimitsu Iinuma, Ikuko Shibuta, Yoshinori Hayashi, Suzuro Hitomi, Koichi Iwata, Masamichi Shinoda

**Affiliations:** 1 Department of Complete Denture Prosthodontics, Nihon University School of Dentistry, Tokyo 101-8310, Japan; deda17003@g.nihon-u.ac.jp (D.I.); deta18004@g.nihon-u.ac.jp (T.O.); desh19025@g.nihon-u.ac.jp (S.F.); iinuma.toshimitsu@nihon-u.ac.jp (T.I.); 2 Department of Physiology, Nihon University School of Dentistry, Tokyo 101-8310, Japan; shibuta.ikuko@nihon-u.ac.jp (I.S.); hayashi.yoshinori@nihon-u.ac.jp (Y.H.); hitomi.suzuro@nihon-u.ac.jp (S.H.); iwata.kouichi@nihon-u.ac.jp (K.I.); shinoda.masamichi@nihon-u.ac.jp (M.S.)

**Keywords:** microglia, M1, M2, senescence-accelerated mice, SAMP8, SAMR1, TNF-α, IL-10, intraoral incision, orofacial mechanical allodynia

## Abstract

Activated microglia involved in the development of orofacial pain hypersensitivity have two major polarization states. The aim of this study was to assess the involvement of the aging-related phenotypic conversion of medullary microglia in the enhancement of intraoral pain sensitivity using senescence-accelerated mice (SAM)-prone/8 (SAMP8) and SAM-resistant/1 (SAMR1) mice. Mechanical head-withdrawal threshold (MHWT) was measured for 21 days post palatal mucosal incision. The number of CD11c-immunoreactive (IR) cells [affective microglia (M1)] and CD163-IR cells [protective microglia (M2)], and tumor-necrosis-factor-α (TNF-α)-IR M1 and interleukin (IL)-10-IR M2 were analyzed via immunohistochemistry on days 3 and 11 following incision. The decrease in MHWT observed following incision was enhanced in SAMP8 mice. M1 levels and the number of TNF-α-IR M1 were increased on day 3 in SAMP8 mice compared with those in SAMR1 mice. On day 11, M1 and M2 activation was observed in both groups, whereas IL-10-IR M2 levels were attenuated in SAMP8 mice, and the number of TNF-α-IR M1 cells increased, compared to those in SAMR1 mice. These results suggest that the mechanical allodynia observed following intraoral injury is potentiated and sustained in SAMP8 mice due to enhancement of TNF-α signaling, M1 activation, and an attenuation of M2 activation accompanying IL-10 release.

## 1. Introduction

Aging is a progressive biological process characterized by neurological dysfunction, such as cognitive impairment [1]. In the central nervous system, amyloid plaque deposition, neurodegeneration, neuropsychiatric conditions, and dementia are associated with aging [2]. Moreover, aging is also known to influence the peripheral nervous system. For example, the excitability of the primary afferent C-fiber is modulated in aged rats [3]. Similarly, the nociceptive neuronal excitability in the lumbar spinal dorsal horn is significantly higher in aged rats than in young rats [4]. Thus, it is possible that aging is also involved in the plastic changes in orofacial nociceptive signal transduction in the medulla; however, the precise molecular mechanisms responsible for the aging-related changes in orofacial nociception remain unknown. Microglial activation is known to modulate nociceptive neuronal excitability in the spinal dorsal horn in response to peripheral pathogenesis, such as sciatic nerve injury [5,6,7,8,9,10]. Microglia are also present in the trigeminal spinal subnucleus caudalis (Vc) and the upper cervical spinal cord (C1/C2). Activation of microglia in the Vc and C1/C2 regions is known to be involved in the pathogenesis of orofacial pain hypersensitivity [11,12,13,14,15]. Microglia have a diverse array of phenotypes and retain the ability to shift function to maintain tissue homeostasis [16]. Affective microglia (M1) are characterized by the production of pro-inflammatory mediators, including interleukin (IL)-1β, tumor necrosis factor-α (TNF-α), and IL-6 [17]. In contrast, protective microglia (M2) are characterized by the production of anti-inflammatory mediators, including IL-4 and IL-10 [18]. Therefore, microglia play dual roles in regulating the onset of inflammation through M1 and then switch to an M2 phenotype to promote healing and repair. However, it is unclear whether age-related neurodegenerative affects the polarity changes of microglia in situations orofacial pathogenesis and if the polarity changes modulate orofacial pain hypersensitivity.

Senescence-accelerated mice (SAM)-prone/8 (SAMP8) show an age-dependent rapid increase in aging markers such as a shortened life span and neuronal histopathology. SAM-resistant/1 (SAMR1) mice are a normal aging control strain for SAMP8 [19,20,21,22]. SAMP8 mice have been used as an age-related neurodegenerative disease model in several studies [23,24,25,26]. The aim of this study was to examine the involvement of the aging-related phenotypic conversion of medullary microglia in the enhancement of palatal mucosal incisional pain sensitivity using SAMP8 and SAMR1 mice.

## 2. Results

### 2.1. Changes in Mechanical Sensitivity Following Incision

**Figure 1:**
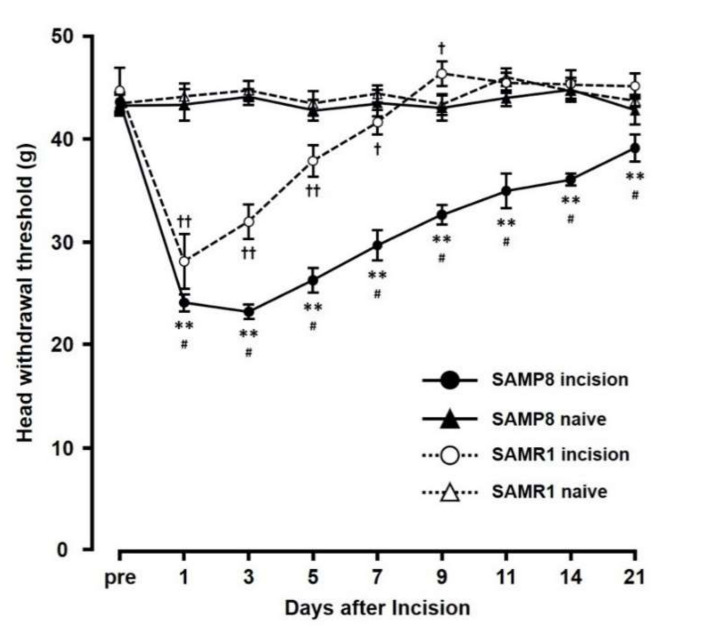
Changes in mechanical head-withdrawal threshold (MHWT) at the incision site over 21 days post palatal mucosal incision. Data represent mean ± SD. (*n* = 6 for SAMP8 incision mice, SAMR1 incision mice, and SAMR1 naive mice, and *n* = 4 SAMP8 for naive mice). Significance was assessed with a two-way ANOVA followed by Bonferroni’s multiple-comparison test. ** *p* < 0.01, SAMP8 incision mice vs. SAMP8 naive mice. † *p* < 0.05, †† *p* < 0.01, SAMR1 incision mice vs. SAMR1 naive mice. # *p* < 0.01, SAMP8 incision mice vs. SAMR1 incision mice.

The mechanical head-withdrawal thresholds (MHWTs) significantly decreased on day 1 through day 7 in the SAMR1 incision mice, and on day 1 through day 21 in the SAMP8 incision mice (*p* < 0.05). On day 3 following incision, MHWTs in both SAMR1 and SAMP8 significantly decreased (SAMR1 incision: 32.0 ± 1.7, SAMR1 naive: 44.7 ± 0.9, SAMP8 incision: 23.2 ± 0.7, SAMP8 naive: 44.1 ± 0.8) compared to that in their respective naive controls (Figure 1). In contrast, by day 11, although MHWT levels in SAMR1 mice had returned to those seen in naive mice, SAMP8 incision mice still showed a significant decrease in MHWT compared to that in naive SAMP8 mice (SAMR1 incision mice (45.5 ± 1.0), SAMR1 naive mice (46.0 ± 0.9), SAMP8 incision mice (35.0 ± 1.7), and SAMP8 naive mice (44.0 ± 0.8)). Overall, comparing the effects in SAMP8 and SAMR1 incision mice, MHWT levels in the SAMP8 incision mice remained low compared with those in SAMR1 incision mice through the experimental period, and did not return to those seen in SAMP8 naive mice.

### 2.2. Microglial Activation in the Vc and C1/C2 Regions

**Figure 2:**
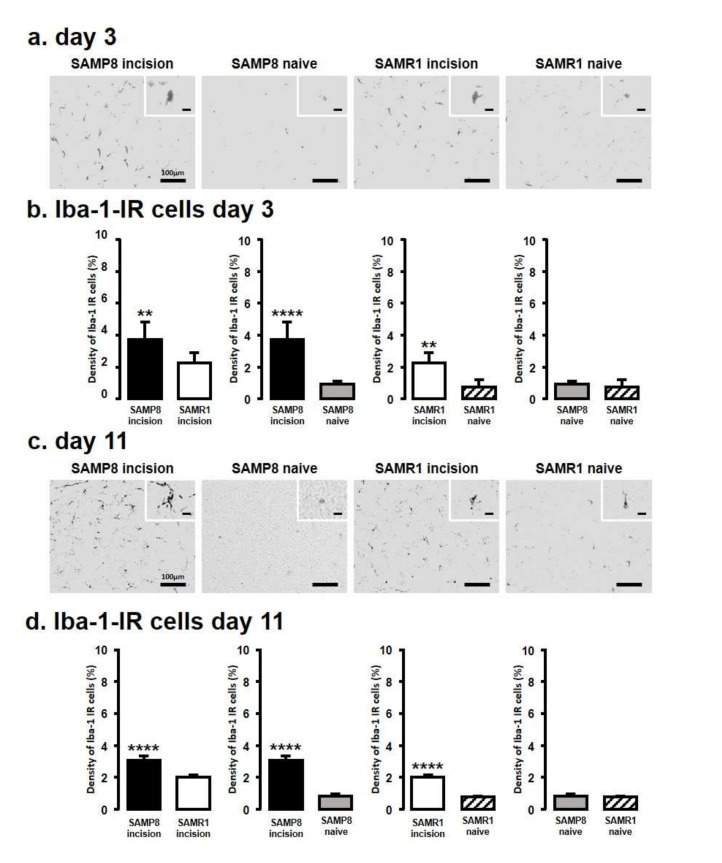
Microglial activation in Vc and C1/C2 regions following palatal mucosal incision. Low and high (inset) magnification fluorescence micrograph of the Iba1-IR cells in the Vc and C1/C2 regions on day 3 (**a**) and day 11 (**c**) after palatal mucosal incision. Scale bar: 100 µm. Scale bar in the inset: 15 µm. Density of the Iba1-IR cells in the Vc and C1/C2 regions on day 3 (**b**) and day 11 (**d**) after palatal mucosal incision. Data represent mean ± SD. (day 3, *n* = 6 for SAMP8 incision mice, SAMR1 incision mice, SAMR1 naive mice, and SAMP8 naive mice; day 11, *n* = 5 for SAMP8 incision mice, SAMR1 incision mice, and SAMR1 naive mice. *n* = 4 for SAMP8 naive mice. ** *p* < 0.01, **** *p* < 0.0001, Student’s *t*-test).

We next assessed Iba1 levels by immunohistochemistry in the Vc and C1/C2 regions innervating the palatal mucosa on days 3 and 11 following the incision (Figure 2). We compared the following pairs in groups: SAMP8-incison vs. SAMR1-incision, SAMP8-incision vs. SAMP8-naive, SAMR1-incision vs. SAMR1-naive, SAMP8-naive vs. SAMR1-naive. On day 3, SAMP8 and SAMR1 mice showed significant increases in the number of Iba1 immunoreactive (IR) cells in the Vc and C1/C2 regions following the incision. The SAMP8 incision mice had significantly more Iba1-IR cells than the SAMR1 incision mice (SAMP8 incision mice: 3.7 ± 1.1, SAMR1 incision mice: 2.3 ± 0.6, SAMP8 naive mice: 0.9 ± 0.2, SAMR1 naive mice: 0.7 ± 0.4) (Figure 2a,b). On day 11 after incision, Iba1-IR cells significantly increased in both the SAMP8 and SAMR1 incision mice; however, the SAMP8 incision mice had significantly more Iba1-IR cells than the SAMR1 incision mice (SAMP8 incision mice: 3.0 ± 0.3, SAMR1 incision mice: 2.0 ± 0.1, SAMP8 naive mice: 0.8 ± 0.1, SAMR1 naive mice: 0.8 ± 0.1) (Figure 2c,d). There were no significant differences in the number of Iba1-IR cells between SAMP8 naive and SAMR1 naive mice on days 3 and 11.

### 2.3. Changes in the Expression of M1 or M2 Microglial Cells in Vc and C1/C2 Regions

**Figure 3:**
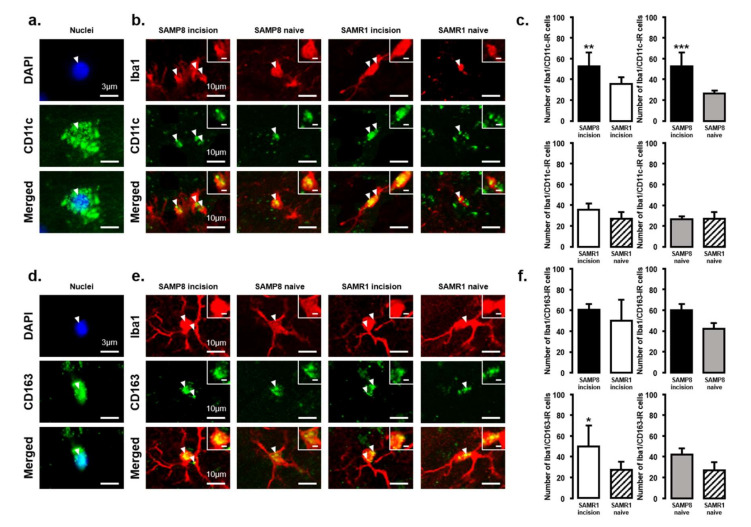
M1/M2 polarization in microglia in the Vc and C1/C2 regions on day 3 after palatal mucosal incision. (**a**) Representative photomicrographs of DAPI-IR cells (blue), CD11c-IR cells (green), and DAPI-IR/CD11c-IR cells in the Vc and C1/C2 regions. Scale bar: 3 µm. (**b**) Low and high (inset) magnification photomicrographs of Iba1-IR cells (red), CD11c-IR cells (green), and Iba1-IR/CD11c-IR cells in the Vc and C1/C2 regions on day 3 after palatal mucosal incision in SAMP8 and SAMR1 mice. The arrow heads indicate Iba1-IR/CD11c-IR cells. Scale bar: 10 µm. Scale bar in the inset: 3 µm. (**c**) The number of Iba1-IR/CD11c-IR cells on day 3 after palatal mucosal incision in SAMP8 and SAMR1 mice. Data represent mean ± SD. (*n* = 6 for SAMP8 incision mice, SAMR1 incision mice, SAMR1 naive mice, and SAMP8 naive mice. ** *p* < 0.01, *** *p* < 0.001, Student’s *t*-test). (**d**) Representative photomicrographs of DAPI-IR cells (blue), CD163-IR cells (green), and DAPI-IR/CD163-IR cells in the Vc and C1/C2 regions. Scale bar: 3 µm. (**e**) Low and high (inset) magnification photomicrographs of Iba1-IR cells (red), CD163-IR cells (green), and Iba1-IR/CD163-IR cells in the Vc and C1/C2 regions on day 3 after palatal mucosal incision. The arrowheads indicate the Iba1-IR/CD163-IR cells. Scale bar: 10 µm. Scale bar in the inset: 3 µm. (**f**) The number of Iba1-IR/CD163-IR cells in the Vc and C1/C2 regions on day 3 after palatal mucosal incision. Data represent mean ± SD. (*n* = 6 for SAMP8 incision mice, SAMR1 incision mice, SAMR1 naive mice, and SAMP8 naive mice. * *p* < 0.05, Student’s *t*-test).

**Figure 4:**
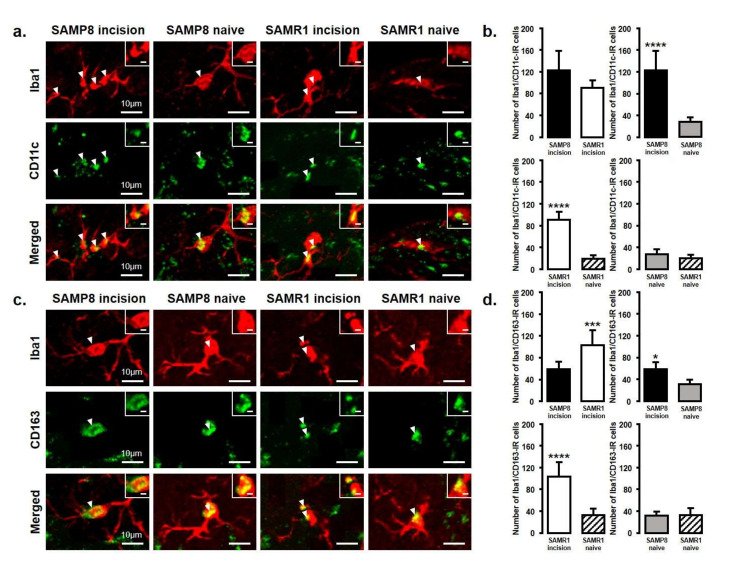
M1/M2 polarization in microglia in the Vc and C1/C2 regions on day 11 after palatal mucosal incision. (**a**) Low and high (inset) magnification photomicrographs of Iba1-IR cells (red), CD11c-IR cells (green), and Iba1-IR/CD11c-IR cells in the Vc and C1/C2 regions on day 11 after palatal mucosal incision in SAMP8 and SAMR1 mice. The arrowheads indicate Iba1-IR/CD11c-IR cells. Scale bar: 10 µm. Scale bar in the inset: 3 µm. (**b**) The number of Iba1-IR/CD11c-IR cells on day 11 after palatal mucosal incision in SAMP8 and SAMR1. Data represent mean ± SD. (*n* = 6 for SAMP8 incision mice, SAMR1 incision mice, SAMR1 naive mice, and SAMP8 naive mice. **** *p* < 0.0001, Student’s *t*-test). (**c**) Low and high (inset) magnification photomicrographs of Iba1-IR cells (red), CD163-IR cells (green), and Iba1-IR/CD163-IR cells in the Vc and C1/C2 regions on day 11 after palatal mucosal incision. The arrowheads indicate the Iba1-IR/CD163-IR cells. Scale bar: 10 µm. Scale bar in the inset: 3 µm. (**d**) The number of Iba1-IR/CD163-IR cells in the Vc and C1/C2 regions on day 11 after palatal mucosal incision. Data represent mean ± SD (*n* = 6 for SAMP8 incision mice, SAMR1 incision mice, SAMR1 naive mice, and SAMP8 naive mice. * *p* < 0.05, *** *p* < 0.001, **** *p* < 0.0001, Student’s *t*-test).

The expression of M1 or M2 phenotype microglial cells in the Vc and C1/C2 regions after palatal mucosal incision (Figure 3 and Figure 4) was examined next. We compared the following pairs in groups: SAMP8 incision vs. SAMR1 incision, SAMP8 incision vs. SAMP8 naive, SAMR1 incision vs. SAMR1 naive, SAMP8 naive vs. SAMR1 naive. Double-immunostaining of the nucleus (DAPI) and M1 (CD11c) or M2 (CD163), clearly demonstrated the specificity of labeling (Figure 3a,d). On day 3, the number of Iba1-IR/CD11c-IR cells following palatal mucosal incision in SAMP8 incision mice significantly increased, but the levels did not change in SAMR1 incision mice (SAMP8 incision mice: 52.7 ± 13.4, SAMR1 incision mice: 35.5 ± 6.2, SAMP8 naive mice: 26.7 ± 2.7, SAMR1 naive mice: 27.2 ± 6.6) (Figure 3b,c). The number of Iba1-IR/CD163-IR cells following palatal mucosal incision significantly increased in SAMR1 incision mice, but similar results were not obtained in SAMP8 incision mice (SAMP8 incision: 60.3 ± 5.8, SAMR1 incision: 49.5 ± 20.4, SAMP8 naive: 42 ± 5.8, SAMR1 naive: 27.2 ± 7.6) (Figure 3e,f). On day 11, both SAMP8 and SAMR1 mice showed a significant increase in Iba1-IR/CD11c-IR cells following palatal mucosal incision compared with each naive mouse strain. Comparing the SAMP8 incision mice to SAMR1 incision mice, there was no difference in the distribution of Iba1/CD11c-IR cells (SAMP8 incision mice: 122.5 ± 35.7, SAMR1 incision mice: 90.2 ± 14.8, SAMP8 naive mice: 27.8 ± 8.6, SAMR1 naive mice: 19.5 ± 6.4) (Figure 4a,b). SAMP8 and SAMR1 mice also showed a significant increase in the number of Iba1-IR/CD163-IR cells following incision, with the SAMR1 incision mice having significantly more Iba1-IR/CD163-IR cells than the SAMP8 incision mice (SAMP8 incision mice: 58.5 ± 12.3, SAMR1 incision mice: 102.3 ± 27.4, SAMP8 naive mice: 31.2 ± 7.5, SAMR1 naive mice: 33.2 ± 11.6) (Figure 4c,d).

### 2.4. Changes in TNF-α or IL-10 Expression in the Vc and C1/C2 Regions

**Figure 5:**
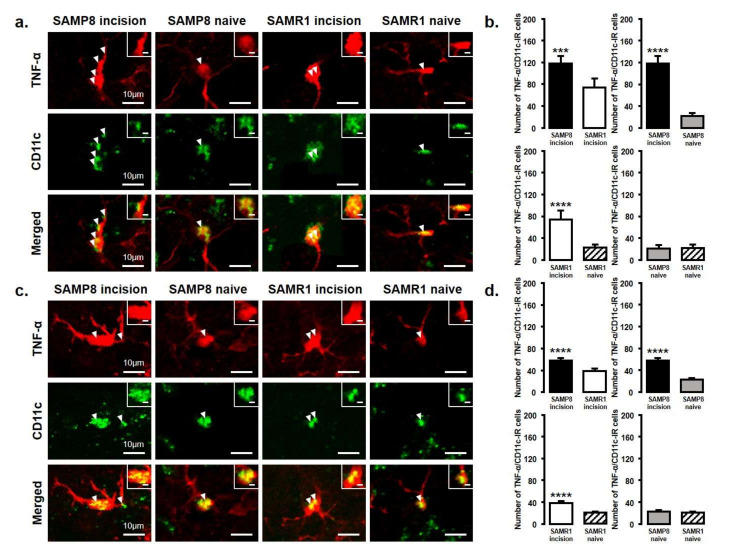
TNF-α released from M1 phenotype microglia in the Vc and C1/C2 regions on day 3 and day 11 after palatal mucosal incision. (**a**) Low and high (inset) magnification photomicrographs of TNF-α-IR cells (red), CD11c-IR cells (green), and TNF-α-IR/CD11c-IR cells in the Vc and C1/C2 regions on day 3 after palatal mucosal incision in SAMP8 and SAMR1 mice. The arrowheads indicate TNF-α-IR/CD11c-IR cells. Scale bar: 10 µm. Scale bar in the inset: 3 µm. (**b**) The number of TNF-α-IR/CD11c-IR cells in the medulla on day 3 after palatal mucosal incision in SAMP8 and SAMR1 mice. Data represent mean ± SD (*n* = 5 for SAMP8 incision mice, SAMR1 incision mice, and SAMR1 naive mice. *n* = 4 for SAMP8 naive mice; *** *p* < 0.001, **** *p* < 0.0001, Student’s *t*-test). (**c**) Low and high (inset) magnification photomicrographs of TNF-α-IR cells (red), CD11c-IR cells (green), and TNF-α-IR/CD11c-IR cells in the Vc and C1/C2 regions on day 11 after palatal mucosal incision in SAMP8 and SAMR1 mice. The arrowheads indicate the TNF-α-IR/CD11c-IR cells. Scale bar: 10 µm. Scale bar in the inset: 3 µm. (**d**) The number of TNF-α-IR/CD11c-IR cells in the medulla on day 11 after palatal mucosal incision in SAMP8 and SAMR1 mice. Data represent mean ± SD (*n* = 5 for SAMP8 incision mice, SAMR1 incision mice, and SAMR1 naive mice. *n* = 4 for SAMP8 naive mice. **** *p* < 0.0001, Student’s *t*-test).

**Figure 6:**
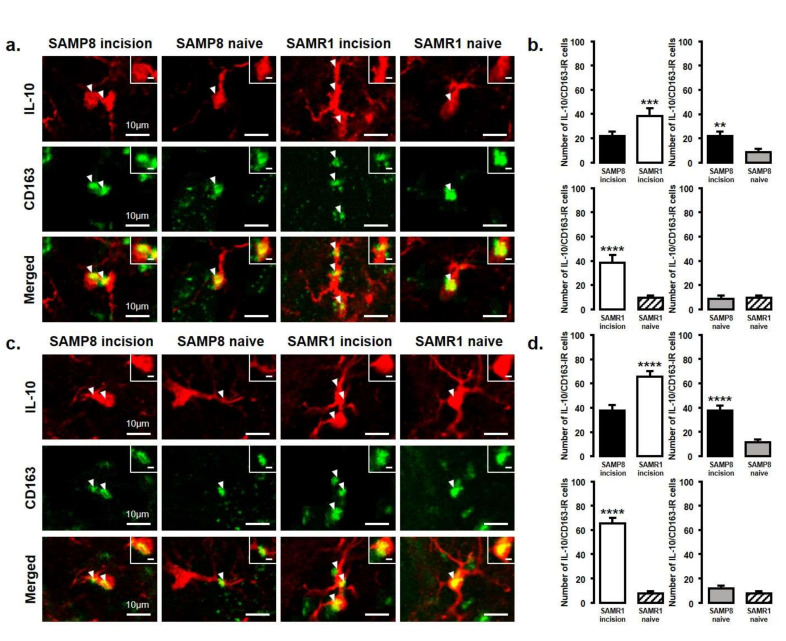
IL-10 released from M2 phenotype microglia in the Vc and C1/C2 regions on day 3 and day 11 after palatal mucosal incision. (**a**) Low and high (inset) magnification photomicrographs of IL-10-IR cells (red), CD163-IR cells (green), and IL-10-IR/CD163-IR cells in the Vc and C1/C2 regions on day 3 after palatal mucosal incision in SAMP8 and SAMR1 mice. The arrowheads indicate IL-10-IR/CD163-IR cells. Scale bar: 10 µm. Scale bar in the inset: 3 µm. (**b**) The number of IL-10-IR/CD163-IR cells in the medulla on day 3 after palatal mucosal incision in SAMP8 and SAMR1 mice. Data represent mean ± SD (*n* = 4 for SAMP8 incision mice, SAMR1 incision mice, SAMR1 naive mice, and SAMP8 naive mice. ** *p* < 0.01, *** *p* < 0.001, **** *p* < 0.0001, Student’s *t*-test). (**c**) Low and high (inset) magnification photomicrographs of IL-10-IR cells (red), CD163-IR cells (green), and IL-10-IR/CD163-IR cells in the Vc and C1/C2 regions on day 11 after palatal mucosal incision in SAMP8 and SAMR1 mice. The arrowheads indicate IL-10-IR/CD163-IR cells. Scale bar: 10 µm. Scale bar in the inset: 3 µm. (**d**) The number of IL-10-IR/CD163-IR cells in the medulla on day 11 after palatal mucosal incision in SAMP8 and SAMR1 mice. Data represent mean ± SD (*n* = 4 for SAMP8 incision mice, SAMR1 incision mice, SAMR1 naive mice, and SAMP8 naive mice. **** *p* < 0.0001, Student’s *t*-test).

The number of TNF-α and IL-10 expressing microglia in the Vc and C1/C2 regions was examined after palatal mucosal incision (Figure 5 and Figure 6). We compared the following pairs in groups: SAMP8-incison vs. SAMR1-incision, SAMP8-incision vs. SAMP8-naive, SAMR1-incision vs. SAMR1-naive, SAMP8-naive vs. SAMR1-naive. The number of TNF-α-IR/CD11c-IR cells was significantly increased in both SAMP8 and SAMR1 mice on days 3 and 11 after incision. In particular, SAMP8 incision mice showed significantly more TNF-α-IR/CD11c-IR cells than SAMR1 incision mice on days 3 and 11 (day 3, SAMP8 incision mice: 117.6 ± 13.5; SAMR1 incision mice: 74 ± 16.7; SAMP8 naive mice: 21.5 ± 5.7; SAMR1 naive mice: 22.4 ± 6.0; day 11, SAMP8 incision mice: 57.2 ± 4.6, SAMR1 incision mice: 37.8 ± 4.4, SAMP8 naive mice: 22.8 ± 2.5, SAMR1 naive mice: 20.4 ± 1.8) (Figure 5a–d).

The number of IL-10-IR/CD163-IR cells was significantly increased in both SAMP8 and SAMR1 mice on days 3 and 11 after incision. Comparing SAMP8 with SAMR1 incision mice, the SAMR1 incision mice showed a significantly higher number of IL-10-IR/CD163-IR cells than the SAMP8 incision mice on days 3 and 11 (day 3, SAMP8 incision mice: 22 ± 3.6; SAMR1 incision mice: 38.3 ± 6.5; SAMP8 naive mice: 8.5 ± 3.1; SAMR1 naive mice: 9.8 ± 2.1; day 11, SAMP8 incision mice: 37.5 ± 4.4 SAMR1 incision mice: 65.3 ± 4.6; SAMP8 naive mice: 11.5 ± 2.4; SAMR1 naive mice: 7.8 ± 1.9) (Figure 6a–d).

### 2.5. Effect of TNF-α, or IL-10 Neutralization or Recombinant IL-10, on Palatal Mucosal Mechanical Hypersensitivities

**Figure 7:**
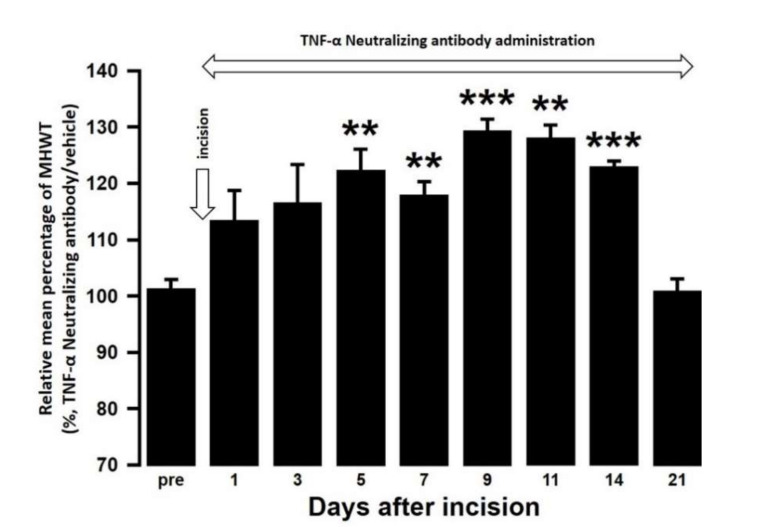
Changes in MHWT in SAMP8 mice treated with either a TNF-α neutralizing antibody or vehicle following palatal mucosal incision. The relative mean percentage MHWT was calculated over 21 days using the following formula (100 × MHWT in SAMP8 mice treated with a TNF-α neutralizing antibody/MHWT in SAMP8 mice treated with vehicle). Data represent mean ± SD (*n* = 5 for SAMP8 incision mice treated with TNF-α neutralizing antibody, and SAMP8 incision mice treated with vehicle. ** *p* < 0.01, *** *p* < 0.001, compared to pre-value; one-way repeated measures ANOVA followed by Tukey’s multiple comparison test).

**Figure 8:**
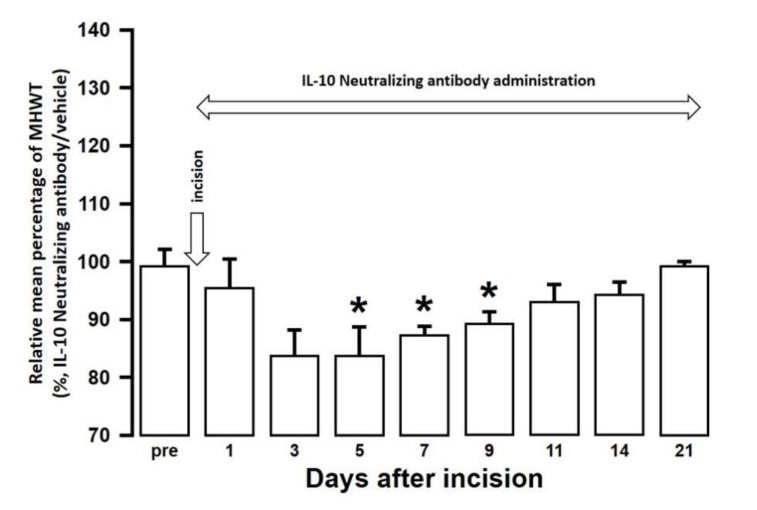
Changes in MHWT in SAMR1 mice treated with either an IL-10 neutralizing antibody or vehicle following palatal mucosal incision. The relative mean percentage MHWT was calculated over 21 days using the following formula (100 × MHWT in SAMR1 mice treated with an IL-10 neutralizing antibody/MHWT in SAMR1 mice treated with vehicle). Data represent mean ± SD (*n* = 5 for SAMR1 incision mice treated with an IL-10 neutralizing antibody, and SAMR1 incision mice treated with vehicle. * *p* < 0.05, compared to pre-value; one-way repeated measures ANOVA followed by Tukey’s multiple comparison test).

**Figure 9:**
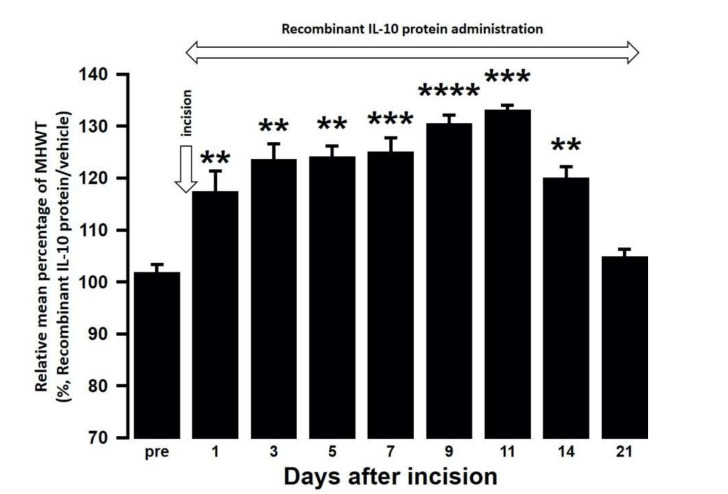
Changes in MHWT in SAMP8 mice treated with either a recombinant IL-10 protein or vehicle following palatal mucosal incision. The relative mean percentage MHWT was calculated over 21 days using the following formula (100 × MHWT in SAMP8 mice treated with recombinant IL-10 protein/MHWT in SAMP8 mice treated with vehicle). Data represent mean ± SD (*n* = 5 for SAMP8 incision mice treated with a recombinant IL-10 protein, and SAMP8 incision mice treated with vehicle. ** *p* < 0.01, *** *p* < 0.001, **** *p* < 0.0001 compared to pre value; one-way repeated measures ANOVA followed by Tukey’s multiple comparison test).

The effect of intra-cisterna magna (ICM) administration of a specific rabbit monoclonal TNF-α neutralizing antibody (10602-R10N1), a specific rat monoclonal IL-10 neutralizing antibody (MAB417), or recombinant rat IL-10 protein (ab269199) on the mechanical hypersensitivities induced by the palatal mucosal incision was examined. The administration of 10602-R10N1 to SAMP8 incision mice significantly suppressed mechanical hypersensitivities from day 5 to day 14 after incision (Figure 7). The administration of MAB417 induced a significant enhancement of mechanical hypersensitivities in SAMR1 incision mice from day 5 to day 9 after incision (Figure 8). The ICM administration of ab269199 significantly suppressed mechanical hypersensitivities in SAMP8 incision mice from day 1 to day 14 after incision (Figure 9).

## 3. Discussion

From day 1 to day 21 after incision, SAMP8 mice developed stronger mechanical allodynia than SAMR1 mice. SAMR1 mice showed recovery of this mechanical allodynia on day 11 after incision. On days 3 and 11, both SAMP8 and SAMR1 mice showed enhanced Iba1 expression in the Vc and C1/C2 regions after incision, and SAMP8 mice also showed significantly abundant Iba1 expression than SAMR1 mice. Aging is known to modulate the release of various cytokines from immunocytes, including microglia, in the spinal dorsal horn [27]. A variety of molecular signaling pathways from glial cells in the spinal dorsal horn play important roles in the modulation of secondary neuronal excitability following peripheral nerve injury [28,29]. In particular, it has been reported that microglial activation is involved in the enhancement of neuronal sensitization relevant to mechanical hypersensitivity under neuropathologic conditions [7]. Interestingly, advancing age is associated with functional changes in microglia, and so is potentially implicated in the changes in nociceptive excitability [30,31]. Together with these reports, our results suggest that aging modulates functional changes in microglia in the Vc and C1/C2 regions following palatal mucosal injury, resulting in an enhancement of mechanical allodynia due to the augmentation of Vc and C1/C2 neuronal hyperexcitability regulated by age-related microglial activation.

It has been reported that CD11c is a surface marker for M1 microglia, and that CD163 is a surface marker for M2 microglia [32,33,34]. When these markers were used to evaluate the microglial polarity in SAMP8 and SAMR1 mice, we found that SAMP8 mice had significantly more M1 microglial cells, but no change was observed in M2 microglial cells, on day 3 after palatal mucosal incision. In addition, SAMP8 mice showed a greater attenuation of M2 microglial levels than SAMR1 mice on day 11 after palatal mucosal incision. In response to the changes in the brain microenvironment caused by aging, microglia can polarize into the proinflammatory M1 phenotype or the anti-inflammatory M2 phenotype [35,36,37,38]. Many studies have reported that the microglia M1 transition is enhanced, but the M2 transition is reduced by aging [39,40,41]. In age-related neurodegenerative diseases, M1 microglial cells are increased, resulting in increased impairment, and M2 microglial cells are reduced, resulting in decreased healing [42]. However, the mechanisms underlying these changes are unclear. Recently, amyloid β (Aβ) plaque deposition in neurons has been reported to be closely related to aging and this deposition causes microglial polarity changes [42,43]. Membrane environmental toxins, such as Aβ aggregates released into the extracellular space from neurons, can directly induce the M1 microglial phenotype. These Aβ plaque aggregates therefore bias microglia towards the M1 phenotype, which persist in later stages of the disease and cause an imbalance in the immune system [42]. SAMP8 mice have been shown to develop senescent amyloidosis due to aging [44]. Aβ-stimulated microglia release ATP which signals through autocrine and paracrine mechanisms via the P2X7 receptor, thereby inducing a polarity change to the pro-inflammatory M1 phenotype microglia that release inflammatory cytokines [45]. Together with these reports, our data also suggest that age-related neuronal alterations modulate microglial polarity, enhancing M1 microglia and attenuating M2 microglia in the Vc and C1/C2 regions following palatal mucosal injury with advancing age. As a result, a sustainable enhancement of palatal mucosal mechanical allodynia occurs due to this imbalance in microglial immune responses in the Vc and C1/C2 regions.

The immune response that arises from the release of proinflammatory and anti-inflammatory cytokines from microglia results in alterations in inter-synaptic signaling and a regulation of the excitability of secondary neurons. On days 3 and 11 after palatal mucosal incision, SAMP8 mice had significantly more TNF-α expressing microglia compared to SAMR1 mice, while SAMR1 mice had significantly more IL-10 expressing M2 microglia than SAMP8 mice. These results indicate that there is a further increase in the proinflammatory cytokine TNF-α, and a weak increase in the anti-inflammatory cytokine IL-10 with advancing age. This is likely due the alteration in microglial polarity, that is, the enhancement of M1 microglia and the reduction of M2 microglia, seen after palatal mucosal incision.

In previous studies, TNF-α released from microglia has been shown to enhance synaptic efficacy by increasing the surface expression of alpha-amino-3-hydroxy-5-methyl-4 isoazolepropionic acid (AMPA) receptors [46]. Furthermore, peripheral tissue inflammation can also induce the TNF-α dependent surface trafficking of Ca2^+^ -permeable AMPA receptors in the spinal dorsal horn [47,48,49]. At the onset of inflammatory pain in the hind paw, TNF-α has been shown to evoke a drastic increase in spontaneous excitatory postsynaptic current frequency in lamina II neurons. TNF-α also increases *N*-methyl-d-aspartate (NMDA) currents in spinal cord lamina II neurons, and this increase is not observed in TNFR1 knockout mice [50]. In addition, TNF-α has also been reported to induce trafficking of NMDA receptors and synaptic plasticity [51]. In this study, ICM administration of a TNF-α neutralizing antibody suppressed the mechanical hypersensitivities in SAMP8 incision mice. Taken together, these results suggest that the persistent M1-derived TNF-α increases due to aging causes surface trafficking of glutamatergic receptors such as AMPA and NMDA in Vc and C1/C2 neurons, which enhances their neuronal excitability and noxious mechanical sensitivity in the palatal mucosa. With respect to the action of TNF-α, it has been reported that TNF-α released from M1 acts on neurons and activates microglia, resulting in a further increase in the release of TNF-α from microglia [52]. Therefore, both the action of TNF-α on neurons and the activation of additional microglia by TNF-α may be involved in mechanical hypersensitivity. However, M1 microglia also release other proinflammatory cytokines such as IL-1β and IL-6. There are also several other surface markers of M1 microglia such as CD40 and CD86 [53]. Therefore, further studies using cytokines other than TNF-α, or other M1 markers are necessary to create a more detailed profile of M1 microglia with advancing age.

The ICM administration of an IL-10 neutralizing antibody enhanced mechanical hypersensitivity in SAMR1 incision mice. In contrast, the ICM administration of a recombinant IL-10 protein suppressed mechanical hypersensitivity in SAMP8 incision mice. On days 3 and 11 after palatal mucosal incision, IL-10 expression was enhanced in M2 microglia in both SAMP8 and SAMR1 mice, but the IL-10 expression levels were lower in SAMP8 mice than in SAMR1 mice. IL-10 is known to be involved in the suppression of mechanical hypersensitivity following plantar incision and spinal nerve ligation [54]. This suppression of mechanical hypersensitivity involves activation of G protein-coupled receptors, such as GPR40, which is expressed in the spinal cord and plays a regulatory role in antinociception via the IL-10/β-endorphin pathway [55]. GPR40 signaling plays an important suppressive role in spinal nociceptive processing after inflammation. For example, GPR40 agonists have been shown to decrease the frequency of spontaneous EPSCs (excitatory postsynaptic currents) in dorsal horn neurons of the spinal cord of inflammatory and neuropathic pain model mice [56]. Another study has reported that the activation of supraspinal GPR40 receptor signaling can regulate the descending pain control system [57]. Therefore, it is possible that the activation of GPR40 signaling involved in antinociception is suppressed by the reduction of IL-10 release from M2 microglia, resulting in an enhancement of mechanical allodynia due to the augmentation of Vc and C1/C2 neuronal excitability associated with age-related neurodegeneration.

TNF-α and IL-10 are reported to regulate the polarity changes of activated microglia to M1 and M2. For instance, TNF-α causes an increase in inflammatory markers, indicating a shift to M1, and a decrease in anti-inflammatory markers, indicating a shift from M2 [52]. In addition, attenuating IL-10 has been shown to promote the polarization of microglia to the M1 phenotype under proinflammatory conditions [58]. Therefore, it is possible that the mechanisms of the microglial polarity change are not independent, but rather are interrelated with each other, and maintaining the balance between M1 and M2 polarization is involved in the suppression of abnormal orofacial pain and delay in healing.

In conclusion, age-related neuroinflammation caused by oral mucosal injury influences the polarity change pattern of activated microglia, which leads to the enhancement of Vc and C1/C2 neuronal excitability by increasing M1-derived TNF-α expression and decreasing IL-10 expression. These novel findings might provide helpful information regarding the mechanism of aging-specific pain hypersensitivity.

## 4. Materials and Methods 

### 4.1. Animals

Male SAMP8 (23 weeks, Japan SLC, Hamamatsu, Japan) and male SAMR1 (23 weeks, Japan SLC) mice weighing 20–30 g were used in all experiments (*n* = 261). Mice were maintained at a controlled temperature (23 °C) under a 12 h light/dark cycle with free access to food and water. This study was approved by the Animal Experimentation Committee at Nihon University School of Dentistry (AP17D010; approval date: 30 June 2017) and was conducted in accordance with the guidelines of the International Association for the Study of Pain [59]. The minimal number of mice required for statistical analysis in the experiments were used. 

### 4.2. Intraoral Incisional Model

Under deep anesthesia induced by an intraperitoneal (IP) injection of butorphanol (5.0 mg/kg; Meiji Seika Pharma, Tokyo, Japan), midazolam (4.0 mg/kg; Sandoz, Tokyo, Japan), and medetomidine (0.75 mg/kg; Zenoaq, Fukushima, Japan), the left palatal mucosa of the mice was incised using a scalpel (depth, 1 mm; length, 5 mm). A sham procedure was performed as a control, which was identical except an incision was made in the left palatal mucosa.

### 4.3. Mechanical Stimulation

Mechanical stimulation was performed after confirming that the mice were maintained at the depth of anesthesia as described above [60,61]. Briefly, the mice were first anesthetized using 2% isoflurane (Mylan, Canonsburg, PA, USA). After stopping the supply of 2% isoflurane, it was confirmed that a normal hindlimb withdrawal reflex could be induced by a noxious pinch to the hind paw while maintaining appropriate breathing and cardiac rhythms. Following this, a mechanical stimulation was applied to a position 1 mm inside from the center of the incision line using an electronic von Frey anesthesiometer (Bioseb, Chaville, France). The mouth was maintained in an open state using a mouth opener under a constantly adjusted depth of anesthesia. The intensity of the mechanical stimulation was gradually increased at a specified speed (0–100 g, 10 g/s, cutoff: 100 g), and the lowest intensity of mechanical stimulus required to induce a head withdrawal reflex was defined as the mechanical head withdrawal threshold (MHWT) [62,63]. Each stimulation interval was 3 min, and the average of three measurements was defined as MHWT in each mouse. MHWT measurements were performed under blinded conditions.

### 4.4. TNF-α and IL-10 Signaling in the Vc and C1/C2 Regions

Mice were anesthetized with an IP injection of butorphanol (5.0 mg/kg), midazolam (4.0 mg/kg), and medetomidine (0.75 mg/kg). A small hole was made in the occipital bone after a small incision was made in the head skin, and a polyethylene tube (0.8 mm in diameter; Natsume, Tokyo, Japan) connected to an osmotic mini pump (0.5 µL/h, Alzet model 2002; Durect Corporation, Cupertino, CA, USA) was inserted into the cisterna magna through the hole [13]. Following this, 10602-R10N1 (500 μg/mL dissolved in 0.01 M PBS, 0.11 μL/h, Sino Biological, Beijing, China), MAB417 (500 μg/mL dissolved in 0.01 M PBS, 0.11 μL/h, R&D Systems, Minneapolis, MN, USA), or ab269199 (0.1 mg/mL, dissolved in 0.01 M PBS, 0.11 μL/h, Abcam, Cambridge, UK) was continually provided via ICM administration for 21 days after the left palatal mucosa incision via the osmotic mini pumps. MHWT was measured daily in each group as described above.

### 4.5. Immunohistochemistry in the Vc and C1/C2 Regions

On day 3 and day 11 after incision to the left palatal mucosa, mice were anesthetized with an IP injection of a mixture composed of butorphanol (5.0 mg/kg), midazolam (4.0 mg/kg), and medetomidine (0.75 mg/kg) and were then perfused with saline and then fixed by perfusing with 4% paraformaldehyde (PFA) in 0.1 M phosphate buffer (PB; pH = 7.4) After dissection, the medulla was fixed in 4% PFA at 4 °C for 24 h. It was then stored in 0.01 M phosphate buffer saline (PBS) containing 20% sucrose for 6 h for cryoprotection. The medulla was cut to a thickness of 30 μm using a freezing microtome (Leica, Tokyo, Japan). After every seven sections, thin tissue slices were collected in 0.01 M PBS. These free-floating tissue sections were rinsed with 0.01 M PBS, and then incubated with a rabbit anti Iba1 polyclonal antibody (1:2000, Wako, Osaka, Japan) at 4 °C for 72 h. The sections were then incubated in 10% normal goat serum (NGS) in PBS for 1.5 h at room temperature (RT; 23 °C). Following this, the sections were incubated at RT for 2 h with a biotinylated goat anti-rabbit IgG (1:600, Vector Laboratories, Burlingame, CA, USA). After washing with 0.01 M PBS, the sections were then incubated at RT for 1 h with a peroxidase-conjugated avidin–biotin complex (1:100, Vector Laboratories, Burlingame, CA, USA). After rinsing in 0.05 M Tris buffer (TB, pH 7.4), the sections were incubated in 0.05 M TB containing 0.035% 3,3′-daiminobenzidine tetrahydrochloride hydrate (DAB, Sigma-Aldrich, St. Louis, MO, USA), 0.2% nickel ammonium sulfate, and 0.05% peroxide for about 5 min. After rinsing in PBS, the sections were sequentially mounted on MAS-coated Superfrost Plus microscope slides (Matsunami, Tokyo, Japan), dehydrated with a series of ethanol (50–100%), and then applied to coverslips. Iba1 expression was analyzed in a square grid (26.7 × 26.7 μm^2^) of the left Vc and C1/C2 regions (360 μm caudal and 360 μm rostral to the obex) that receive afferents from the second branch of the trigeminal nerve, which innervates the left palatal mucosa. The Iba1 immunoreactive area was measured using a computer-assisted imaging analysis system (Image J 1.37v; NIH, Bethesda, MD, USA). Additionally, we performed immunohistochemistry without the Iba1 primary antibody for each group of mice and confirmed that there was no immunostaining (data not shown).

The sections were also incubated with a rabbit polyclonal Iba1 antibody (1:2000, 019-19741; Wako, Osaka, Japan) and an Armenian hamster monoclonal CD11c antibody (1:1000, ab33483; Abcam, Cambridge, UK) to identify M1 microglia; with a goat polyclonal Iba1 antibody (1:500, ab5076; Abcam, Cambridge, UK) and a rabbit recombinant monoclonal CD163 antibody (1:1000, ab182422; Abcam, Cambridge, UK) to identify M2 microglia; with an Armenian hamster monoclonal CD11c antibody (1:1000, ab33483; Abcam, Cambridge, UK) and a rabbit polyclonal TNF-α antibody (1:200, ab6671; Abcam, Cambridge, UK) to identify TNF-α positive M1 microglia; or with a rabbit recombinant monoclonal CD163 antibody (1:1000, ab182422; Abcam, Cambridge, UK) and a rat monoclonal IL-10 antibody (1:300, ab33471; Abcam, Cambridge, UK) to identify IL-10 positive M2 microglia. Following this primary incubation, the sections were incubated with either an Alexa Fluor 568-conjugated goat anti-rabbit IgG (1:1,000; Life Technologies, Waltham, MA, USA), an Alexa Fluor 488-conjugated goat-anti-Armenian hamster IgG (1:1000; Abcam, Cambridge, UK), an Alexa Fluor 568-conjugated donkey-anti-goat IgG (1:1000; Life Technologies, Waltham, MA, USA), an Alexa Fluor 568-conjugated donkey-anti-rat IgG (1:1000; Abcam, Cambridge, UK), an Alexa Fluor 488-conjugated donkey-anti-rabbit IgG (1:1000; Invitrogen, Waltham, MA, USA), an Alexa Fluor 488-conjugated goat-anti-Armenian hamster IgG (1:1000; Abcam, Cambridge, UK), or an Alexa Fluor 488-conjugated donkey-anti-rabbit IgG (1:1000; Invitrogen, Carlsbad, CA, USA) and DAPI (1:5000, D9564; Sigma-Aldrich, St. Louis, MO, USA) in 0.01 M PBS for 2 h at RT, as appropriate. Following rinsing with 0.01 M PBS, the sections were mounted in mounting medium (Thermo Fisher Scientific, Waltham, MA, USA).

Immunofluorescent signals for Iba1 and CD11c, Iba1 and CD163, CD11c, TNF-α, CD163, and IL-10 immune products were visualized using a BZ-9000 system (Keyence, Tokyo, Japan). The signal in a square grid (26.7 × 26.7 μm^2^) in the Vc and C1/C2 regions was then quantified using a computer-assisted imaging analysis system (Image J 1.37v; NIH, Bethesda, MD, USA).

### 4.6. Statistical Analysis

The data in this study are presented as mean ± standard deviation (SD). One-way or two-way repeated measures analysis of variance (ANOVA) followed by Tukey’s multiple comparison test, or a Student’s *t-*test were used for the assessments of behavioral testing or immunohistochemistry analysis, where appropriate. Statistical significance was determined to be *p* < 0.05.

## Abbreviations

SAMP8	Senescence accelerated mice prone 8
SAMR1	Senescence accelerated mice resistant 1
M1	Affective microglia
M2	Protective microglia
MHWT	mechanical head withdrawal threshold
TNF-α	tumor-necrosis-factor-α
IL-10	interleukin-10
ICM	intra–cisterna magna

## References

[1] Niraula A., Sheridan J.F., Godbout J.P. (2017). Microglia Priming with Aging and Stress. Neuropsychopharmacology.

[2] Wyss-Coray T. (2016). Ageing, neurodegeneration and brain rejuvenation. Nature.

[3] Taguchi T., Ota H., Matsuda T., Murase S., Mizumura K. (2010). Cutaneous C-fiber nociceptor responses and nociceptive behaviors in aged Sprague–Dawley rats. Pain.

[4] Iwata K., Fukuoka T., Kondo E., Tsuboi Y., Tashiro A., Noguchi K., Masuda Y., Morimoto T., Kanda K. (2002). Plastic changes in nociceptive transmission of the rat spinal cord with advancing age. J. Neurophysiol..

[5] Peritore A.F., Siracusa R., Fusco R., Gugliandolo E., D’Amico R., Cordaro M., Crupi R., Genovese T., Impellizzeri D., Cuzzocrea S. (2020). Ultramicronized Palmitoylethanolamide and paracetamol, a new association to relieve hyperalgesia and pain in a sciatic nerve injury model in rat. Int. J. Mol. Sci..

[6] Xiang Y., Liu T., Yang H., Gao F., Xiang H., Manyande A., Tian Y., Tian X. (2015). NRG1-ErbB signalling promotes microglia activation contributing to incision-induced mechanical allodynia. Eur. J. Pain.

[7] Ledeboer A., Sloane E.M., Milligan E.D., Frank M.G., Mahony J.H., Maier S.F., Watkins L.R. (2005). Minocycline attenuates mechanical allodynia and proinflammatory cytokine expression in rat models of pain facilitation. Pain.

[8] Svensson C.I., Fitzsimmons B., Azizi S., Powell H.C., Hua X.Y., Yaksh T.L. (2005). Spinal p38beta isoform mediates tissue injury-induced hyperalgesia and spinal sensitization. J. Neurochem..

[9] Obata H., Eisenach J.C., Hussain H., Bynum T., Vincler M. (2006). Spinal glial activation contributes to postoperative mechanical hypersensitivity in the rat. J. Pain.

[10] D’Amico R., Impellizzeri D., Cuzzocrea S., Di Paola R. (2020). ALIAmides Update: Palmitoylethanolamide and its formulations on management of peripheral neuropathic pain. Int. J. Mol. Sci..

[11] Okada-Ogawa A., Suzuki I., Sessle B.J., Chiang C.Y., Salter M.W., Dostrovsky J.O., Tsuboi Y., Kondo M., Kitagawa J., Kobayashi A. (2009). Astroglia in medullary dorsal horn (trigeminal spinal subnucleus caudalis) are involved in trigeminal neuropathic pain mechanisms. J. Neurosci..

[12] Sago T., Ono K., Harano N., Furuta-Hidaka K., Hitomi S., Nunomaki M., Yoshida M., Shiiba S., Nakanishi O., Matsuo K. (2012). Distinct time courses of microglial and astrocytic hyperactivation and the glial contribution to pain hypersensitivity in a facial cancer model. Brain Res..

[13] Tamagawa T., Shinoda M., Honda K., Furukawa A., Kaji K., Nagashima H., Akasaka R., Chen J., Sessle B.J., Yonehara Y. (2016). Involvement of microglial P2Y12 signaling in tongue cancer Pain. J. Dent. Res..

[14] Shinoda M., Kubo A., Hayashi Y., Iwata K. (2019). Peripheral and central mechanisms of persistent orofacial Pain. Front. Neurosci..

[15] Old E.A., Clark A.K., Malcangio M. (2015). The role of glia in the spinal cord in neuropathic and inflammatory pain. Handb. Exp. Pharmacol..

[16] Orihuela R., McPherson C.A., Harry G.J. (2016). Microglial M1/M2 polarization and metabolic states. Br. J. Pharmacol..

[17] Kalkman H.O., Feuerbach D. (2016). Antidepressant therapies inhibit inflammation and microglial M1-polarization. Pharmacol. Ther..

[18] Genovese T., Esposito E., Mazzon E., Di Paola R., Caminiti R., Bramanti P., Cappelani A., Cuzzocrea S. (2009). Absence of endogenous interleukin-10 enhances secondary inflammatory process after spinal cord compression injury in mice. J. Neurochem..

[19] Takeda T., Hosokawa M., Higuchi K. (1997). Senescence-accelerated mouse (SAM): A novel murine model of senescence. Exp. Gerontol..

[20] Takeda T. (2009). Senescence-accelerated mouse (SAM) with special references to neurodegeneration models, SAMP8 and SAMP10 mice. Neurochem. Res..

[21] Miyamoto M., Kiyota Y., Yamazaki N., Nagaoka A., Matsuo T., Nagawa Y., Takeda T. (1986). Age-related changes in learning and memory in the senescence-accelerated mouse (SAM). Physiol. Behav..

[22] Butterfield D.A., Poon H.F. (2005). The senescence-accelerated prone mouse (SAMP8): A model of age-related cognitive decline with relevance to alterations of the gene expression and protein abnormalities in Alzheimer’s disease. Exp. Gerontol..

[23] Jiang J., Liu G., Shi S., Li Y., Li Z. (2019). Effects of manual acupuncture combined with donepezil in a mouse model of Alzheimer’s disease. Acupunct. Med..

[24] Chen W., Liang T., Zuo W., Wu X., Shen Z., Wang F., Li C., Zheng Y., Peng G. (2018). Neuroprotective effect of 1-Deoxynojirimycin on cognitive impairment, beta-amyloid deposition, and neuroinflammation in the SAMP8 mice. Biomed. Pharmacother..

[25] Cheng X.R., Zhou W.X., Zhang Y.X., Zhou D.S., Yang R.F., Chen L.F. (2007). Differential gene expression profiles in the hippocampus of senescence-accelerated mouse. Neurobiol. Aging.

[26] Fernandez-Gomez F.J., Munoz-Delgado E., Montenegro M.F., Campoy F.J., Vidal C.J., Jordan J. (2010). Cholinesterase activity in brain of senescence-accelerated-resistant mouse SAMR1 and its variation in brain of senescence-accelerated-prone mouse SAMP8. J. Neurosci. Res..

[27] Rea I.M., Gibson D.S., McGilligan V., McNerlan S.E., Alexander H.D., Ross O.A. (2018). Age and Age-Related Diseases: Role of Inflammation Triggers and Cytokines. Front. Immunol..

[28] Milligan E.D., Watkins L.R. (2009). Pathological and protective roles of glia in chronic pain. Nat. Rev. Neurosci..

[29] Wilkerson J.L., Milligan E.D. (2011). The Central Role of Glia in Pathological Pain and the Potential of Targeting the Cannabinoid 2 Receptor for Pain Relief. ISRN Anesthesiol..

[30] Norden D.M., Godbout J.P. (2013). Review: Microglia of the aged brain: Primed to be activated and resistant to regulation. Neuropathol. Appl. Neurobiol..

[31] Paladini A., Fusco M., Coaccioli S., Skaper S.D., Varrassi G. (2015). Chronic pain in the elderly: The case for new therapeutic strategies. Pain Physician.

[32] Xu Y., Qian L., Zong G., Ma K., Zhu X., Zhang H., Li N., Yang Q., Bai H., Ben J. (2012). Class A scavenger receptor promotes cerebral ischemic injury by pivoting microglia/macrophage polarization. Neuroscience.

[33] Ono Y., Nagai M., Yoshino O., Koga K., Nawaz A., Hatta H., Nishizono H., Izumi G., Nakashima A., Imura J. (2018). CD11c+ M1-like macrophages (MPhis) but not CD206+ M2-like MPhi are involved in folliculogenesis in mice ovary. Sci. Rep..

[34] Zhang Z., Zhang Z.Y., Schittenhelm J., Wu Y., Meyermann R., Schluesener H.J. (2011). Parenchymal accumulation of CD163+ macrophages/microglia in multiple sclerosis brains. J. Neuroimmunol..

[35] Wang G., Zhou Y., Wang Y., Li D., Liu J., Zhang F. (2019). Age-associated dopaminergic neuron loss and midbrain glia cell phenotypic polarization. Neuroscience.

[36] Zhang Q., Lu Y., Bian H., Guo L., Zhu H. (2017). Activation of the alpha7 nicotinic receptor promotes lipopolysaccharide-induced conversion of M1 microglia to M2. Am. J. Transl. Res..

[37] Ma Y., Wang J., Wang Y., Yang G.Y. (2017). The biphasic function of microglia in ischemic stroke. Prog. Neurobiol..

[38] Xu S., Zhu W., Shao M., Zhang F., Guo J., Xu H., Jiang J., Ma X., Xia X., Zhi X. (2018). Ecto-5’-nucleotidase (CD73) attenuates inflammation after spinal cord injury by promoting macrophages/microglia M2 polarization in mice. J. Neuroinflamm..

[39] Huntula S., Saegusa H., Wang X., Zong S., Tanabe T. (2019). Involvement of N-type Ca(2+) channel in microglial activation and its implications to aging-induced exaggerated cytokine response. Cell Calcium.

[40] Yao K., Zhao Y.F. (2018). Aging modulates microglia phenotypes in neuroinflammation of MPTP-PD mice. Exp. Gerontol..

[41] Li M.D., Burns T.C., Kumar S., Morgan A.A., Sloan S.A., Palmer T.D. (2015). Aging-like changes in the transcriptome of irradiated microglia. Glia.

[42] Tang Y., Le W. (2016). Differential Roles of M1 and M2 Microglia in Neurodegenerative Diseases. Mol. Neurobiol..

[43] Silva C.S., Eira J., Ribeiro C.A., Oliveira A., Sousa M.M., Cardoso I., Liz M.A. (2017). Transthyretin neuroprotection in Alzheimer’s disease is dependent on proteolysis. Neurobiol. Aging.

[44] Del Valle J., Duran-Vilaregut J., Manich G., Pallas M., Camins A., Vilaplana J., Pelegri C. (2011). Cerebral amyloid angiopathy, blood-brain barrier disruption and amyloid accumulation in SAMP8 mice. Neurodegener. Dis..

[45] Chiozzi P., Sarti A.C., Sanz J.M., Giuliani A.L., Adinolfi E., Vultaggio-Poma V., Falzoni S., Di Virgilio F. (2019). Amyloid beta-dependent mitochondrial toxicity in mouse microglia requires P2X7 receptor expression and is prevented by nimodipine. Sci. Rep..

[46] Beattie E.C., Stellwagen D., Morishita W., Bresnahan J.C., Ha B.K., Von Zastrow M., Beattie M.S., Malenka R.C. (2002). Control of synaptic strength by glial TNFalpha. Science.

[47] Choi J.I., Svensson C.I., Koehrn F.J., Bhuskute A., Sorkin L.S. (2010). Peripheral inflammation induces tumor necrosis factor dependent AMPA receptor trafficking and Akt phosphorylation in spinal cord in addition to pain behavior. Pain.

[48] Wigerblad G., Huie J.R., Yin H.Z., Leinders M., Pritchard R.A., Koehrn F.J., Xiao W.H., Bennett G.J., Huganir R.L., Ferguson A.R. (2017). Inflammation-induced GluA1 trafficking and membrane insertion of Ca(2+) permeable AMPA receptors in dorsal horn neurons is dependent on spinal tumor necrosis factor, PI3 kinase and protein kinase A. Exp. Neurol..

[49] Gary D.S., Bruce-Keller A.J., Kindy M.S., Mattson M.P. (1998). Ischemic and excitotoxic brain injury is enhanced in mice lacking the p55 tumor necrosis factor receptor. J. Cereb. Blood Flow Metab..

[50] Zhang L., Berta T., Xu Z.Z., Liu T., Park J.Y., Ji R.R. (2011). TNF-alpha contributes to spinal cord synaptic plasticity and inflammatory pain: Distinct role of TNF receptor subtypes 1 and 2. Pain.

[51] Wheeler D., Knapp E., Bandaru V.V., Wang Y., Knorr D., Poirier C., Mattson M.P., Geiger J.D., Haughey N.J. (2009). Tumor necrosis factor-alpha-induced neutral sphingomyelinase-2 modulates synaptic plasticity by controlling the membrane insertion of NMDA receptors. J. Neurochem..

[52] Jin M.M., Wang F., Qi D., Liu W.W., Gu C., Mao C.J., Yang Y.P., Zhao Z., Hu L.F., Liu C.F. (2018). A Critical role of autophagy in regulating microglia polarization in neurodegeneration. Front. Aging Neurosci..

[53] Zhou T., Huang Z., Sun X., Zhu X., Zhou L., Li M., Cheng B., Liu X., He C. (2017). Microglia Polarization with M1/M2 Phenotype Changes in rd1 Mouse Model of Retinal Degeneration. Front. Neuroanat..

[54] Dai W.J., Sun J.L., Li C., Mao W., Huang Y.K., Zhao Z.Q., Zhang Y.Q., Lu N. (2019). Involvement of Interleukin-10 in Analgesia of Electroacupuncture on Incision Pain. Evid. Based Complement. Alternat. Med..

[55] Mao X.F., Wu H.Y., Tang X.Q., Ali U., Liu H., Wang Y.X. (2019). Activation of GPR40 produces mechanical antiallodynia via the spinal glial interleukin-10/beta-endorphin pathway. J. Neuroinflamm..

[56] Karki P., Kurihara T., Nakamachi T., Watanabe J., Asada T., Oyoshi T., Shioda S., Yoshimura M., Arita K., Miyata A. (2015). Attenuation of inflammatory and neuropathic pain behaviors in mice through activation of free fatty acid receptor GPR40. Mol. Pain.

[57] Nakamoto K., Nishinaka T., Sato N., Aizawa F., Yamashita T., Mankura M., Koyama Y., Kasuya F., Tokuyama S. (2015). The activation of supraspinal GPR40/FFA1 receptor signalling regulates the descending pain control system. Br. J. Pharmacol..

[58] Laffer B., Bauer D., Wasmuth S., Busch M., Jalilvand T.V., Thanos S., Meyer Zu Horste G., Loser K., Langmann T., Heiligenhaus A. (2019). Loss of IL-10 Promotes Differentiation of Microglia to a M1 Phenotype. Front. Cell. Neurosci..

[59] Zimmermann M. (1983). Ethical guidelines for investigations of experimental pain in conscious animals. Pain.

[60] Shimizu K., Asano M., Kitagawa J., Ogiso B., Ren K., Oki H., Matsumoto M., Iwata K. (2006). Phosphorylation of extracellular signal-regulated kinase in medullary and upper cervical cord neurons following noxious tooth pulp stimulation. Brain Res..

[61] Nagashima H., Shinoda M., Honda K., Kamio N., Hasuike A., Sugano N., Arai Y., Sato S., Iwata K. (2017). CXCR4 signaling contributes to alveolar bone resorption in Porphyromonas gingivalis-induced periodontitis in mice. J. Oral Sci..

[62] Urata K., Shinoda M., Ikutame D., Iinuma T., Iwata K. (2018). Involvement of transient receptor potential vanilloid 2 in intra-oral incisional pain. Oral Dis..

[63] Urata K., Shinoda M., Honda K., Lee J., Maruno M., Ito R., Gionhaku N., Iwata K. (2015). Involvement of TRPV1 and TRPA1 in incisional intraoral and extraoral pain. J. Dent. Res..

